# Ultra-Thin Pyrocarbon Films as a Versatile Coating Material

**DOI:** 10.1186/s11671-017-1896-0

**Published:** 2017-02-16

**Authors:** Tommi Kaplas, Polina Kuzhir

**Affiliations:** 1 Institute of Photonics, University of Eastern Finland, Yliopistokatu 7, 80101 Joensuu, Finland; 2 Research Institute for Nuclear Problems, 11 Bobrujskaya Str., Minsk, 220030 Belarus; 3 Ryazan State RadioEngineering University, 59/1 Gagarina Street, Ryazan, 390005 Russia

**Keywords:** Pyrolytic carbon, Pyrolyzed photoresist, Nanographite film, Chemical vapor deposition

## Abstract

The properties and synthesis procedures of the nanometrically thin pyrolyzed photoresist films (PPF) and the pyrolytic carbon films (PCF) were compared, and a number of similarities were found. Closer examination showed that the optical properties of these films are almost identical; however, the DC resistance of PPF is about three times higher than that of PCF. Moreover, we observed that the wettability of amorphous PPF and PCF was almost comparable to crystalline graphite. Potential applications executed by utilizing the small difference in the synthesis procedure of these two materials are suggested.

## Background

Graphitic carbon is a versatile material that has been widely studied throughout the centuries. Especially, fullerenes, carbon nanotubes, and recently graphene materials have received wide attention. At the same time, amorphous carbon remains somewhat overshadowed by these crystalline materials. One can attribute this to the incomparability of the vast majority of electrical and optical properties of amorphous carbon films and their crystalline counterpart (e.g., graphene) [1, 2]. However, amorphous carbon films have one significant advantage over crystalline graphitic carbon, that is, the production cost and simplicity. These properties are themselves a reasonable justification for the preference of utilization of these materials in the industrial sector.

Two well-known nano-graphitic carbon materials are a pyrolyzed photoresist film (PPF) [3, 4] and a pyrolytic carbon film (PCF) [1, 5]. Although in contrast to graphite and graphene the crystallization of these materials is very low [1, 3, 5, 6], the simplicity of the deposition of these films on various surfaces makes them a good competitor for graphene.

As the name describes, the PPF is a carbon-based photoresist film that has been pyrolyzed at high temperature [3, 4]. During the pyrolysis, the resist solvents are evaporated and remaining carbon atoms are hybridized by sp^2^ and sp^3^ bonds [3]. The noteworthy factor here is that the PPF is fabricated from photoresist, while PCF is conventionally fabricated by chemical vapor deposition (CVD) via gaseous hydrocarbon pyrolysis in the temperature range of 1000 °C and above [6, 7]. In the lower temperature regime, i.e., at around 1000 °C, the nano-graphitic carbon is typically very amorphous. However, by increasing the temperature, the degree of crystallization increases as well [8, 9]. Eventually above 2000 °C, the CVD procedure results in the highly oriented pyrolytic graphite [8, 9]. However, since only a few substrates can hold such an extreme synthesis temperature, the deposition temperature of PCF rarely exceeds 1200 °C.

The interesting aspect for both PCF and PPF is that they can be deposited as an ultra-thin film on a dielectric and a semiconducting substrate [1, 4]. Although PCF and PPF are seemingly very similar materials, there are still some small differences present. In this paper, we give an insight to the synthesis and properties of these two ultra-thin carbon materials. A remarkable fact is the influence of the small difference in the synthesis procedure of these two materials (PPF is fabricated by photoresist precursor and PCF in homogeneous hydrocarbon CVD) on the range of their applicability. As part of the discussion of the properties of these materials, recommendations for their potential use are given. Learning the small differences between these films can easily help one to find the most suitable technique to fabricate carbon layers to suite the desired purpose.

## Methods

Experimentally, a PPF and a PCF can be synthetized on any substrate that withstands the 1100 °C processing temperature. For this particular experiment, we used the silica substrates (later, for demonstration purposes in the “Discussion” section, also silicon and oxidized silicon substrates were also used).

A PPF was fabricated by spin coating the substrate by the carbon-based photoresist (nLOF-AZ2070 diluted with AZ ebr solvent with a 1:4 ratio). After the substrate is coated by a resist layer, it is baked on a hot plate (110 °C/1 min) and then pyrolyzed in a CVD system. The thickness ratio of the original photoresist film and the PPF is 1:10, i.e., the thickness of a 300-nm-thick photoresist film resulted in a 30 (±3)-nm-thick PPF. The thickness of the PPF film can be varied by changing the thickness of the resist film [4]. In contrast to the PPF, a PCF is fabricated by using gaseous CH_4_ as a carbon precursor. Thickness of the PCF is controlled by methane concentration in the process (see, e.g., [5]). For our experiment, we used a CH_4_:H_2_ ratio of 4:1 at about 32 mBar which in turn resulted in a PCF with thickness of 30 (±3) nm.

Our conventional hot wall CVD consists of a vacuum chamber, computerized mass flow controllers, and gas lines for hydrogen, methane, and nitrogen. Before each process, the chamber is pumped down for 1 h to ensure proper vacuum. In order to make the comparison of the PPF and PCF more transparent, the processing temperatures were the same for PPF and PCF. More precisely, the chamber is heated to 700 °C at 20 °C/min and then to 1100 °C at 10 °C/min. The maximum temperature of 1100 °C is kept for 5 min, and then, the chamber is cooled down to 700 °C in 1.5 h; the rest of the cooling is done overnight. Since the photoresist acts as the carbon precursor in PPF, the sample is heated and cooled in the hydrogen atmosphere. Heating is done in 5 sccm H_2_ flow (0.2 mBar) and cooling, in contrary, in static H_2_ atmosphere of 5 mBar. Alternatively, the PCF is done on a substrate by first heating the chamber to 700 °C in H_2_ flow (5 sccm) and then injecting the CH_4_:H_2_ gas mixture into the chamber. Thereafter, at 700 °C, the CH_4_:H_2_ gas mixture is replaced by H_2_ (static 5 mBar) in which the rest of the cooling took place. Since PCF is grown on both sides of the sample, the back side carbon is removed by the oxygen plasma (100 W/20 sccm/2 min).

## Results

**Fig. 1:**
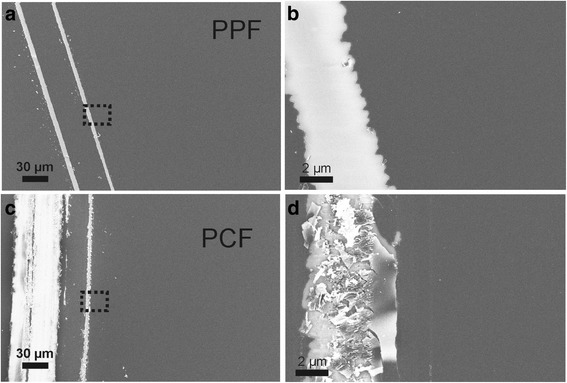
Scanning electron microscope figure of **a**, **b** the PPF and **c**, **d** the PCF on silica substrates. **a**, **c** Low magnification image shows that the PPF and the PCF are very uniform around a scratch made by a scalpel. This uniformity goes throughout the sample surface. **b**, **d** High magnification SEM image reveals that PPF and PCF rip out from the substrate by slightly different manner

Scanning electron microscope (SEM - Leo 1550 Gemini) images of the PPF and the PCF on silica substrates are shown in Fig. 1. Both of the films are very uniform throughout the sample surface. The thickness of the synthetized films was 30 ± 3 nm in both cases (measured by stylus profiler Dektak 150) without any drastic differences over the substrate surface. In Fig. 1, one can observe a scratch in the PPF and the PCF. It is noteworthy that the PPF has been scratched out fully while the PCF has left behind ribbons of the film. This indicates that the PPF could have a better adhesion to the silica substrate in comparison to the PCF. A simple Scotch tape experiment reveals that the PPF is well attached to the surface and it cannot be removed by the tape but instead the PCF can be removed partially or fully from the substrate surface with the Scotch tape.

Electrical DC resistivity was measured by a conventional four-point-probe technique using 2.5-V source voltage and 1.5-mm equal probe spacing (with Signatone S-1160 probe station and self-made setup). The sheet resistance (*R*_*s*_) was defined by the well-known Eq. 1 [10]:

1 $$ {R}_s=\frac{\pi}{ \ln (2)}\cdot \frac{V}{I}, $$

where *V* is the potential difference of two inner probes and *I* is the current of outer probes. The sheet resistance was 3580 (±310) Ω/sq and 1200 (±42) Ω/sq for the PPF and the PCF, respectively. Thus, by multiplying with the film thickness one receives, the resistivity for the PPF is 110 × 10^−5^ Ωcm and 36 × 10^−5^ Ωcm for the PCF. The strong difference of the resistivity of the films is peculiar but can be explained by the difference in the synthesis of the films. Since the carbon precursor of the PPF is a photoresist, one can expect that some part of the resist contaminants remains inside the PPF. These defects can increase electron scattering in the PPF and increase DC resistivity. On the other hand, the process of PCF consists of hydrogen and methane only and thus the PCF is expected to have only carbon in it. Although the PPF has higher resistivity than the PCF, in comparison to non-doped graphene (*R*_*s*_ ~2 kΩ/sq with thickness of 0.34 nm [11] ≥0.68 × 10^−5^ Ωcm), the resistivity of the PCF is still roughly two orders of magnitude higher. However, the PCF can be deposited on a catalytic copper surface, which will increase the crystallinity of the PCF. This was observed to decrease DC resistance down to around 8 × 10^−5^ Ωcm [12]. Moreover, despite the PPF showing higher DC resistivity, it could perform better as an electrochemical contact material [1, 4].

**Fig. 2:**
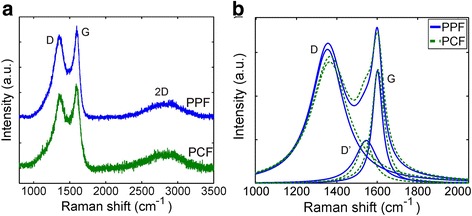
**a** The measured Raman spectrum of PPF and PCF shows dominating D and G peaks. **b** Closer analysis of spectra reveals the D’ peak

Raman spectrums were measured by Renishaw inVia Raman microscope using 514-nm excitation wavelength (Fig. 2). Raman characterization is a conventional technique to recognize different carbon allotropes. In graphite, there are typically three dominating peaks in the Raman spectrum. Namely, those are G (“graphite”) at 1582 cm^−1^, D (“disorder”) at around 1350 cm^−1^, and 2D peak at around 2700 cm^−1^. In highly crystalline graphite, the disorder-induced D peak is often negligible but sharp G and 2D peaks dominate the spectrum [8, 9]. When the graphitic material is highly amorphous like the PPF and the PCF, the magnitude of D peak increases and G mode is broadened [9, 13]. Moreover, since the 2D peak is related to the symmetrical graphite lattice vibrations requiring two breathing mode phonons in opposite phase [14], this peak almost disappears. Also in between D and G peaks, a D’ peak appears (located around 1550 cm^−1^). This peak is related to the amount of amorphous carbon in the material [15].

More careful analysis reveals that there are practically no differences in the Raman spectrum of the PPF and the PCF. However, although the films are very amorphous, the existence of D and G peaks reveal that the material is a nano-graphitic material [9, 13, 15].

**Fig. 3:**
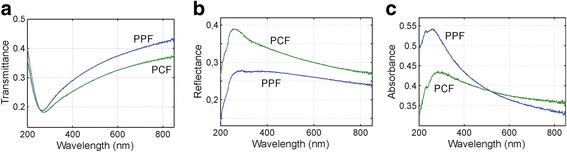
**a** Transmittance, **b** reflectance, and **c** absorbance spectra of PPF and PCF

Since the PPF and the PCF are semitransparent, it is reasonable to measure the optical transmittance, reflectance, and absorbance spectra of the films. Optical absorption was measured with Perkin Elmer Lambda-9 spectrophotometer with integrating sphere at 200–850-nm spectral range. Measured spectra are demonstrated in Fig. 3 that shows the linear optical properties of the films in the spectral range from 200 to 850 nm. Interestingly the PPF is absorbing less efficiently at near infrared in comparison to the PCF. However, the absorption of the PPF increases, and at the ultraviolet range, the absorbance of the PPF is about 10% than the one of PCF. Moreover, the wide absorption peaks are located at 262 nm in the PPF and 284 in the PCF.

Optical absorption in graphitic carbon is governed by the pi-electrons [16], and thus, it is expected that the optical absorption is similar in all graphitic carbon materials with dominating sp^2^ hybridization. In crystalline graphene, the linear electron band structure can be well described as a constant at NIR but slightly changes due to the M-saddle point absorption at UV (the absorption peak maximum is located at around 260 nm) [17]. In that sense, both of the carbon films are somewhat close to the optical properties of graphene/graphite [16, 17]. However, despite the absorption spectra of the PCF and the PPF somewhat resembling the graphene’s absorption spectrum, it is reasonable to remember that due to the amorphous nature of the PPF and the PCF, those films have no well-specified electron band structure.

**Fig. 4:**
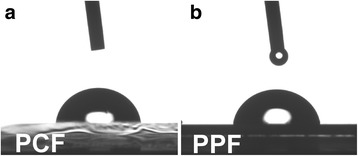
PCF and PPF are both hydrophilic surfaces. The contact angle of water droplet on **a** PCF and **b** PPF is almost the same (~85°) and somewhat comparable to that of crystalline graphite [18]

Surface wettability was measured by using KSV CAM 200 contact angle measurement setup, with 9 μl water droplet at room temperature. Figure 4 shows a water droplet on the PCF (contact angle is 86.0 ± 0.9°) and the PPF (contact angle is 84.5 ± 0.5°) surfaces. The contact angle of the PCF and the PPF surfaces is below 90° indicating slight hydrophilicity and is lower in comparison to hydrophobic graphene with the contact angle as high as 100° [18]. However, the contact angle of amorphous PCF and PPF is only a bit lower in comparison to that of crystalline graphite (~90°) [18].

## Discussion

**Fig. 5:**
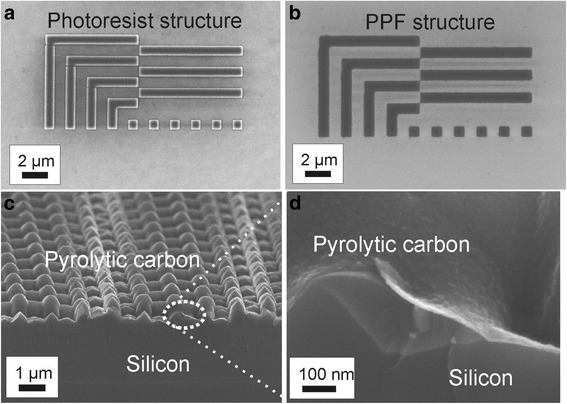
A patterned photoresist structure **a** before and **b** after pyrolysis in 1100 °C temperature. The PPF structure on oxidized (300 nm) silicon holds its form in high temperature. **c**, **d** The PCF can be easily grown on arbitrary shape structures. Still resulting uniform and conformal carbon coating

The PPF and the PCF both appear to be very uniform and optically comparable but with strong difference in electrical resistance. Although the properties of these films are similar and those samples look almost identical, the main difference of the films is the synthesis precursor. It is reasonable to remember that PPF is fabricated by using conventional photoresist as a carbon precursor. This will restrict the substrate for PPF coating to flat, spin-coatable substrates. However, since the precursor is photoresist, it is possible to first pattern the resist film and then pyrolyze it in order to produce different PPF structures. In Fig. 5, an electron beam (Vistec Ebeam EBPG5000) patterned micro-structure of photoresist is shown before and after pyrolysis. This method offers a very simple and straightforward technique to make, e.g., PPF microelectrodes or grating structures on dielectrics or semiconducting materials. Since PPF is a conductor, those structures could be used in, e.g., electrical graphitic contacts or micro- and nanoelectromechanical systems (MEMS and NEMS) [19].

Since the PCF grows in the homogenous CVD process, the film appears as a uniform coating throughout the surface despite the surface texture. More precisely, the PCF can be easily grown on an arbitrarily shaped substrate/structure where the sample size is restricted only by the CVD chamber. In Fig. 5, one can observe about 10-nm-thick PCF grown on a patterned silicon substrate (see detailed growth parameters in [5]). In spite of the grating structure, the carbon film is continuous and homogeneous. Since carbon is often considered as a bio-compatible material, such a hydrophilic coating could offer a nice platform, e.g., for biological studies. Also, since PCF is a conductive material, this film could be used as a conductive layer when electroplating metals (this will be reported elsewhere) or a contact material for semiconductors [20]. Furthermore, because of strong optical absorption in PCF, an ultra-thin PCF was recently used to increase the absorption of black silicon [21].

**Fig. 6:**
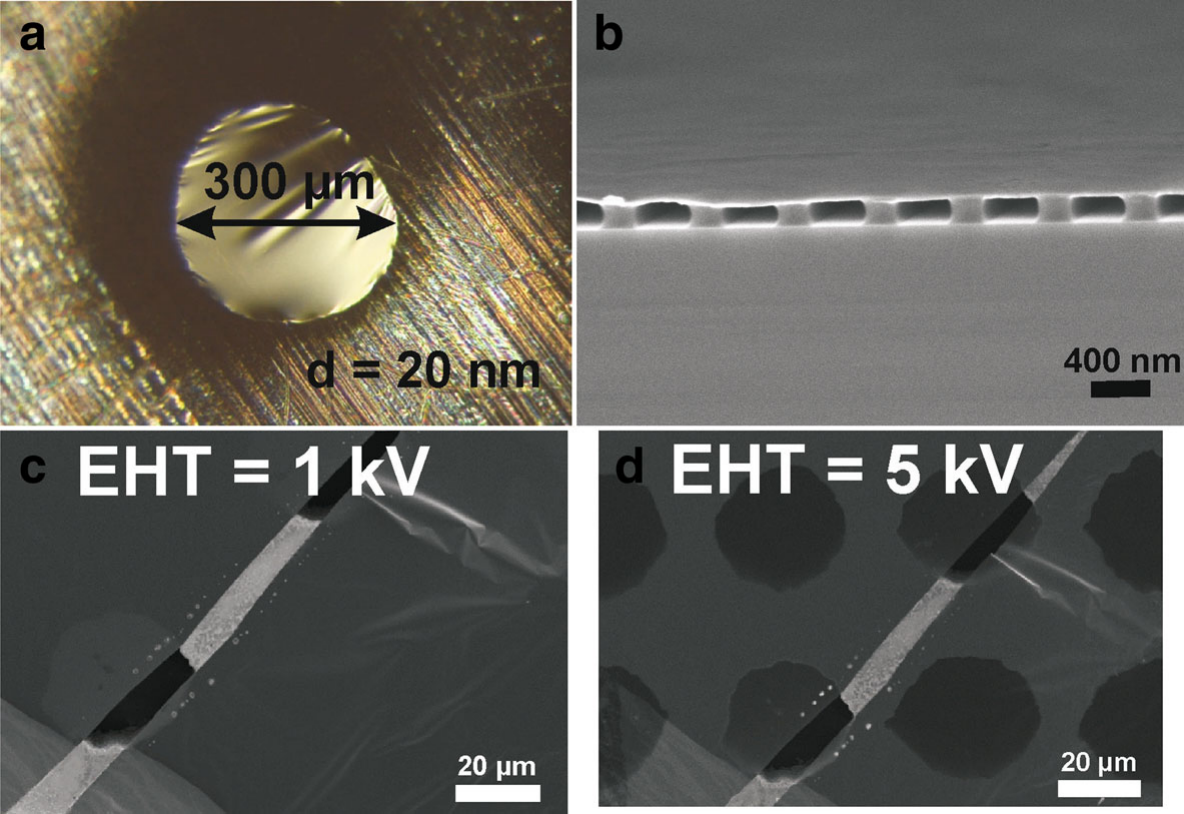
A 30-nm-thick PCF can be transferred, e.g., **a** on a pin hole with 300 μm diameter or **b** on a grating structure without supporting polymer layer. Also with 1-kV extra high tension (EHT), the film is nontransparent, but when EHT is increased to 5 kV, the film becomes semitransparent

As was mentioned earlier in the “Material Synthesis” section, the PCF can be detached from the silica substrate rather easily. We have observed that sometimes the surface tension of water detaches the PCF from the silica surface. In such a case, the PCF will stay floating on the water surface and it can be collected on various surfaces. In Fig. 5, PCF sample has been deposited on various surfaces after removing it from the silica substrate. In Fig. 5a, the film is deposited on a pin hole in a copper foil. Although the film thickness is only 30 nm, the film stays together over a hole that is 300 μm in diameter. PCF can be also deposited on a grating structure (Fig. 6b), where it can serve a purpose, e.g., as a resonator membrane. Moreover, because carbon atoms are very light, an ultra-thin PCF is semitransparent for electrons. In Fig. 6c, d, SEM images of the PCF is shown with low (1 kV) and high (5 kV) acceleration voltages, respectively. This kind of a carbon membrane could be applied to various systems including, e.g., photoelectron spectroscopy or MEMS/NEMS. It is noteworthy that 30-nm-thick PCF is strong enough that it can be transferred without supporting polymer layer, which is required for graphene transfer [11].

Moreover, the robustness of the PCF is indicated by a recent explosive electron emission study where the PCF was suggested to be a coating material for the development of a hybrid cathode, which was characterized by low plasma expansion velocity [22]. The PCF coating allowed generation of powerful microwave pulses of long duration, up to microseconds along with high efficiency (due to the low velocity scatter electrons emitted from the cathode) [22].

## Conclusions

Physical properties of two slightly different nanoscale carbon films were compared. The PPF and the PCF were grown on a silica substrate in very similar processes, which allowed us to compare differences of these films in an unbiased manner. Overall, the Raman spectra and linear optical absorption of PPF and PCF were almost identical for both of the films, while the electrical DC resistivity was observed to be higher for the PPF then for PCF. Moreover, as an alternative material for graphene, either film could be applied in experiments where very thin carbon layer is found to be beneficial.

## Abbreviations

CVD: Chemical vapor deposition
DC: Direct current
MEMS: Microelectromechanical system
NEMS: Nanoelectromechanical system
PCF: Pyrolytic carbon film
PPF: Pyrolyzed photoresist film
